# The Protective Effect of Insulin on Rat Cortical Neurons in Oxidative Stress and Its Dependence on the Modulation of Akt, GSK-3beta, ERK1/2, and AMPK Activities

**DOI:** 10.3390/ijms20153702

**Published:** 2019-07-29

**Authors:** Irina O. Zakharova, Tatiana V. Sokolova, Liubov V. Bayunova, Inna I. Zorina, Maria P. Rychkova, Alexander O. Shpakov, Natalia F. Avrova

**Affiliations:** Department of Molecular Endocrinology and Neurochemistry, I.M. Sechenov Institute of Evolutionary Physiology and Biochemistry of the Russian Academy of Sciences, Thorez avenue, 44, Saint-Petersburg 194223, Russia

**Keywords:** insulin, cortical neurons, oxidative stress, protection, protein kinases, Bax/Bcl-2 ratio, mitochondrial membrane potential

## Abstract

Insulin is a promising drug for the treatment of diseases associated with brain damage. However, the mechanism of its neuroprotective action is far from being understood. Our aim was to study the insulin-induced protection of cortical neurons in oxidative stress and its mechanism. Immunoblotting, flow cytometry, colorimetric, and fluorometric techniques were used. The insulin neuroprotection was shown to depend on insulin concentration in the nanomolar range. Insulin decreased the reactive oxygen species formation in neurons. The insulin-induced modulation of various protein kinase activities was studied at eight time-points after neuronal exposure to prooxidant (hydrogen peroxide). In prooxidant-exposed neurons, insulin increased the phosphorylation of GSK-3beta at Ser^9^ (thus inactivating it), which resulted from Akt activation. Insulin activated ERK1/2 in neurons 5–30 min after cell exposure to prooxidant. Hydrogen peroxide markedly activated AMPK, while it was for the first time shown that insulin inhibited it in neurons at periods of the most pronounced activation by prooxidant. Insulin normalized Bax/Bcl-2 ratio and mitochondrial membrane potential in neurons in oxidative stress. The inhibitors of the PI3K/Akt and MEK1/2/ERK1/2 signaling pathways and the AMPK activator reduced the neuroprotective effect of insulin. Thus, the protective action of insulin on cortical neurons in oxidative stress appear to be realized to a large extent through activation of Akt and ERK1/2, GSK-3beta inactivation, and inhibition of AMPK activity increased by neuronal exposure to prooxidant.

## 1. Introduction

The excessive activation of free radical reactions in brain nerve cells is one of the main causes of their injury and death in various diseases concerned with brain damage [1,2,3]. Such natural antioxidants as carnosine, flavonoids, components of vitamin E, gangliosides, insulin, and others exert their protective and antioxidant action on neurons and other cells mainly by modulating the activity of signaling pathways.

Insulin increases the viability of nerve cells. It is considered promising as a cure for the neurodegenerative, ischemic, diabetic, and other diseases associated with brain injury, intranasal administration of insulin being especially effective in the treatment of these diseases [4,5,6]. It is of interest that there are many common features of Alzheimer’s disease and Type 2 diabetes mellitus; according to some authors, Alzheimer’s disease may be characterized as Type 3 diabetes mellitus [7]. However, the mechanism of the neuroprotective effect of insulin is far from being understood. Meanwhile, effective use in clinical practice of insulin and other natural bioactive substances with antioxidant activity still requires a more in-depth understanding of the way they increase the viability of neurons and other cells modulating the activity of various signaling pathways. In particular, such studies may identify the compounds which markedly enhance the protective effect of each other.

The insulin-induced activation of phosphoinositide 3-kinase (PI3K)/protein kinase B (Akt) signaling pathway increases the viability of various cells, including nerve cells [8,9,10,11,12]. It may result in the activation of the CREB (cAMP response element binding protein) transcription factor and in the increase of the level of antiapoptotic protein Bcl-2 in the cells [13,14]. The pro- and antiapoptotic proteins are important regulators of apoptosis. If the ratio Bax/Bcl-2 is high, mitochondria initiate the program of cell death. Besides, the activation of Akt by some of the neuroprotectors was shown to lead to the inactivation of glycogen synthase kinase 3-beta (GSK-3beta) [15]. GSK-3beta has regulatory effects on mitochondrial biogenesis, bioenergetics, permeability, and apoptosis. The use of GSK-3beta inhibitors as drugs may be promising in management of many diseases including Alzheimer’s and Parkinson’s diseases [16]. 

It seems of importance to elucidate the insulin effect on Bax/Bcl-2 ratio and the mechanism of its modulation of GSK-3beta activity in the nerve cells. However, the data obtained in many cases appear to be incomplete or contradictory. For example, the mechanism of insulin-induced inactivation of GSK-3beta in nerve cells is not clear. According to Duarte and coauthors [17] insulin does not change the level of pGSK-3beta (Ser^9^) in brain cortical neurons subjected to prooxidant action or in control neurons. The authors suggest quite another mechanism of GSK-3beta inhibition by insulin [17]. The data showing the insulin effect on Bax/Bcl-2 ratio in neurons subjected to toxic agents appear to be incomplete. Insulin was shown to prevent the diminution of Bcl-2 level in cortical neurons after their exposure to prooxidants [17] and to decrease Bax/Bcl-2 ratio in SH-5Y cells [11]. But, the effect of insulin on Bax/Bcl-2 ratio and the dynamics of the changes of this ratio in the course of oxidative stress development were not studied in primary cultures of nerve cells. The data showing the IGF effects on the Bax/Bcl-2 ratio in nerve cells exposed to toxic agents appear to be contradictory [18,19].

Protein kinases, such as AMP-activated protein kinase (AMPK), may also be involved in the realization of protective effect of insulin on nerve cells. Inhibition of this signaling pathway appears to be one of the anabolic stimuli of insulin. Administration of insulin to free moving fasting mice reduced AMPK activity in all hypothalamic regions but not in brain cortex of these animals [20]. The results of this work [20] and other data provide evidence that insulin is a potent anorexigenic hormone. But it is not clear how insulin modulates the activity of this protein kinase in nerve cells not belonging to hypothalamic brain regions (either control, or under conditions of oxidative stress). It would also be of interest to know if the protective effect of insulin depends on the modulation of this protein kinase activity.

When studying the contribution of insulin-induced modulation of various protein kinase activities to its protective effect, it is of importance not to forget about extracellular signal-regulated protein kinase (ERK1/2). There are numerous data showing the insulin-induced activation of ERK1/2 in extraneural cells. At the same time, the data showing the insulin effect on ERK1/2 activity in brain nerve cells are contradictory [17,21,22].

In our work the effect of insulin on the activity of various protein kinases was determined by immunoblotting in the dynamics of oxidative stress development (at eight time intervals after induction of oxidative stress). The aim of the present work is to study the protective and antioxidant effects of insulin, to find out how the protective effect of insulin depends on its concentration, and to study the modulatory effect of insulin on the activity of protein kinase B (Akt), ERK1/2, GSK-3beta, and AMPK, on the ratio of proapoptotic to antiapoptotic proteins (Bax/Bcl-2) in the course of oxidative stress development in brain cortical neurons exposed to hydrogen peroxide. The protective effects of insulin on brain cortical neurons in oxidative stress were shown to be realized to great extent through its activation of Akt, inactivation of GSK-3beta as a result of its phosphorylation at Ser^9^, normalization of Bax/Bcl-2 ratio, early activation of ERK1/2, and inhibition of AMPK markedly activated by the prooxidant.

## 2. Results

### 2.1. Insulin Increases Viability and Prevents Apoptotic Death of Rat Brain Cortical Neurons Exposed to Hydrogen Peroxide 

**Figure 1:**
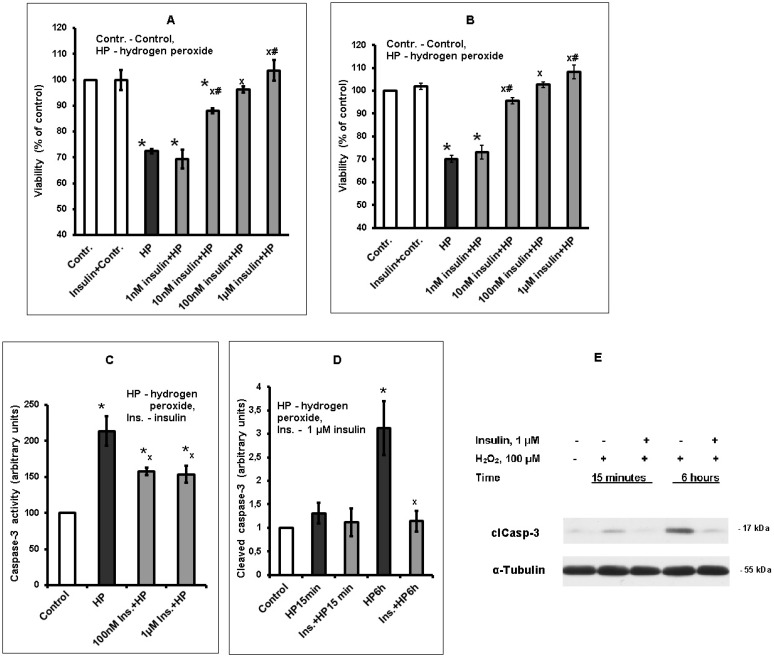
The effect of insulin and hydrogen peroxide on cortical neuron viability, caspase-3 activity and cleaved caspase-3 level in these cells. **A** and **B**. The results of one typical experiment (from 5 replicates) are given as means ± SEM of 2–3 parallel determinations. The cell viability was estimated by MTT method. Cortical neurons were preincubated for 1 h with various insulin concentrations, then neurons were exposed for 6 h to 50 µM (**A**) or 100 µM (**B**–**E**) hydrogen peroxide. The differences are significant according to one-way ANOVA followed by Tukey’s multiple comparison test as compared: *—to control, ^x^—to the effect of hydrogen peroxide alone, ^#^—to the effect of 1 nM insulin and hydrogen peroxide, ^##^—to the effect of 10 nM insulin and hydrogen peroxide, *p* < 0.01 in all cases. **C**—caspase-3 activity, **D**—cleaved caspase level. The data of 5–6 experiments are given as means ± SEM. The differences are significant according to one-way ANOVA as compared: *—to control, ^x^—to the effect of hydrogen peroxide alone, **C**—*p* < 0.05, **D**—*p* < 0.001. **E**. Immunoblots of cleaved caspase-3, showing the effect of insulin on the level of 17–19 kDa fragment 15 min and 6 h after exposure of neurons to hydrogen peroxide.

Insulin was shown to increase the viability of cultured rat brain cortical neurons exposed to hydrogen peroxide in a large range of concentrations (from 10 nM to 10 µM), We used 100 µM (sometimes 50 µM) hydrogen peroxide as a prooxidant because it is a natural prooxidant; its content in the brain may be increased in ischemic or other unfavorable conditions. The protective effect of insulin was found to depend on its concentration (1 nM < 10 nM < 100 nM or 1 µM insulin), as it is seen from the results of the typical experiments made using 50 µM and 100 µM of hydrogen peroxide as a prooxidant (Figure 1A,B, respectively). The difference in the effects of 100 nM and 1 µM insulin was not revealed.

In order to compare the results of all experiments made, the percent of rat brain cortical neurons, whose death was prevented by preincubation of cells with insulin prior to prooxidant application (rescue rates) was calculated. In the experiments using 50 µM hydrogen peroxide as prooxidant, the rescue rates for 1 nM, 10 nM, 100 nM, and 1 µM insulin (*n* = 5) were found to be 10.4 ± 6.6%, 44.5 ± 5.2%, 61.8 ± 10.8%, and 79.5 ± 12.2%, respectively. These data provide evidence that the protective effect of 10 nM, 100 nM, and 1 µM insulin is significant (*p* < 0.02 by Student’s *t* test) in contrast to the effect of 1 nM insulin (*p* > 0.05). The rescue rate of 10 nM insulin was higher than the rescue rate of 1 nM insulin (*p* < 0.01 by Student’s *t* test), while the rescue rate of 1 µM insulin was more pronounced than that of 10 nM insulin (*p* < 0.05 by Student’s *t* test). No difference was revealed in the protective effect of 100 nM and 1 µM insulin (*p* > 0.05).

In order to show that apoptotic death occurred as a result of cortical neuron exposure to 100 µM hydrogen peroxide, we determined the effect of this prooxidant on caspase-3 activity and on the level of cleaved caspase 3 in the samples. Preincubation of cortical neurons with insulin was shown to diminish both the increase of caspase-3 activity and of the level of cleaved caspase-3 induced by the prooxidant. The effect of insulin and hydrogen peroxide on the caspase-3 activity in cortical neurons is shown in Figure 1C. We monitored apoptosis by measuring the fluorescence generated due to hydrolysis of a fluorogenic caspase-3 substrate acetyl- Asp-Glu-Val-Asp-7-amido-4-methylcoumarin (Ac-DEVD-AMC) added to the assay medium, using CASP-3-F kit (Sigma-Aldrich, USA). The exposure to hydrogen peroxide for 6 h markedly increases the activity of caspase-3 in cortical neurons, while preincubation with 100 nM or 1 µM insulin significantly diminishes it. On average, preincubation with 1 µM insulin diminished the increase of caspase-3 activity induced by hydrogen peroxide (*n* = 5) by 53.1 ± 7.1%.

In supplemental materials Figure S1 shows how we determined the molecular weight of caspase 3 cleaved fragment and of alpha-tubulin using protein standards with known molecular weights. 

The effect of insulin and prooxidant on the cleaved caspase-3 level was also studied (Figure 1D and E). Neuron exposure to hydrogen peroxide for 6 h elevated several times the level of cleaved caspase-3 (Figure 1D), as shown by the increase of its 17–19 kDa fragment content on immunoblots (Figure 1E), while preincubation with 1 µM insulin markedly and significantly diminished it. The detection of cleaved caspase-3 is considered a reliable marker for cells which are dying, or have died by apoptosis. Thus, the results obtained provide evidence that cortical neurons exposed to hydrogen peroxide undergo death by apoptosis, while insulin is able to reduce the number of apoptotic cells. 

### 2.2. Insulin Diminishes Reactive Oxygen Species (ROS) Formation Induced by Hydrogen Peroxide in Cortical Neurons 

**Figure 2:**
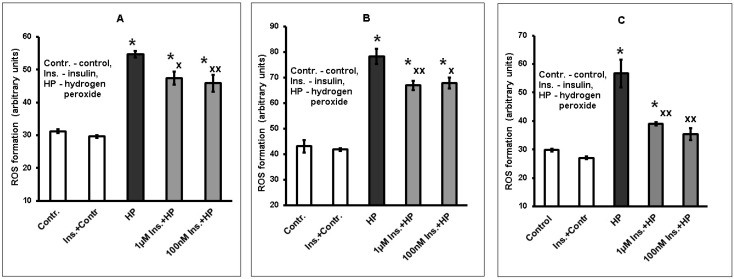
Insulin decreases ROS formation in cortical neuron exposed to hydrogen peroxide. The results of one typical experiment (from five replicates) are given as means ± SEM of 4–6 parallel determinations. Brain cortical neurons were incubated with 1 µM or 100 nM insulin for 1 h (**A**), 5 h (**B**) or 20 h (**C**), then the cells were exposed to 100 µM hydrogen peroxide. ROS accumulation was estimated using dichlorofluorescin diacetate. The differences are significant according to one-way ANOVA as compared: *—to the control values, *p* < 0.01; ^x^ and ^xx^—to the effect of hydrogen peroxide alone, ^x^ *p* < 0.05, ^xx^ *p* < 0.01.

Preincubation with 1 µM and 100 nM insulin lowers the ROS formation elicited by hydrogen peroxide in brain cortical neurons. A significant effect was achieved with insulin preincubation for 1 h (Figure 2A) or 5 h (Figure 2B). If insulin preincubation was conducted for 20 h, the decrease of ROS formation due to insulin effect was more pronounced (Figure 2C). No significant difference was revealed in the effects of 1 µM and 100 nM insulin on ROS formation in neurons.

### 2.3. The Protective and Antioxidant Effects of Insulin on Brain Cortical Neurons Exposed to Hydrogen Peroxide are Diminished in the Presence of Inhibitors of PI3K/Akt and MEK1/2/ERK1/2 Signaling Pathways

**Figure 3:**
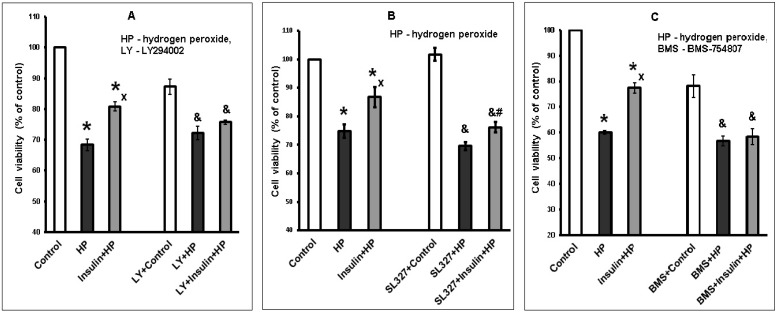
The protective effect of insulin against hydrogen peroxide-induced cortical neuron death is diminished (or abolished) in the presence of LY294002, SL327, and BMS-754807. The results of one typical experiment (from six replicates) are given as means ± SEM of 3–4 parallel determinations. Cortical neurons were incubated: **A**—with 30 µM LY294002, **B**—with 10 µM SL327, **C**—with 1 µM BMS-754807 or without the inhibitors. Then neurons were incubated with 1 µM insulin and exposed to 100 µM hydrogen peroxide. The cell viability was estimated by MTT method. The differences are significant according to one-way ANOVA as compared: *—to control values, *p* < 0.01, ^x^ and ^xx^—to the effect of hydrogen peroxide alone, ^x^ *p* < 0.05, ^xx^ *p* < 0.01, ^&^—to the effect of inhibitor and control, *p* < 0.05, **^#^**—to the effect of inhibitor and hydrogen peroxide.

Activation of Akt is considered to play an important role in the influence of insulin on cell viability and metabolism. Other protein kinases may also contribute to its protective and antioxidant effects. We studied the effect of the inhibitors of signaling pathways PI3K/Akt and MEK1/2/ERK1/2 (LY294002 and SL327, respectively) on the ability of insulin to enhance the viability of neurons and to decrease ROS formation in them. The protective effect of insulin on cortical neurons exposed to hydrogen peroxide is diminished in the presence of LY294002, SL327, and BMS-754807 (the inhibitor of insulin and IGF-1 receptor tyrosine kinase) as one can see looking at the data obtained in a typical experiments (Figure 3A–C). 

The data of all experiments performed on the protective effect of insulin were expressed as rescue rates and are shown in Table 1. The rescue rates of insulin diminished in the presence in all samples of LY294002 or SL327 from 41.65 ± 2.4% to 20.8 ± 6.3% and 19.9 ± 5.6% (*p* < 0.01) respectively (Table 1). These results suggest that activation of both Akt, and ERK1/2 contributes to the protective effect of insulin. It was confirmed that neuroprotection by insulin depends exclusively on its interaction with its receptors. Thus, in the presence of BMS-754807 (the inhibitor of insulin and IGF-1 receptor tyrosine kinase), the rescue rates of insulin were lessened to an insignificant value (Table 1).

**Figure 4:**
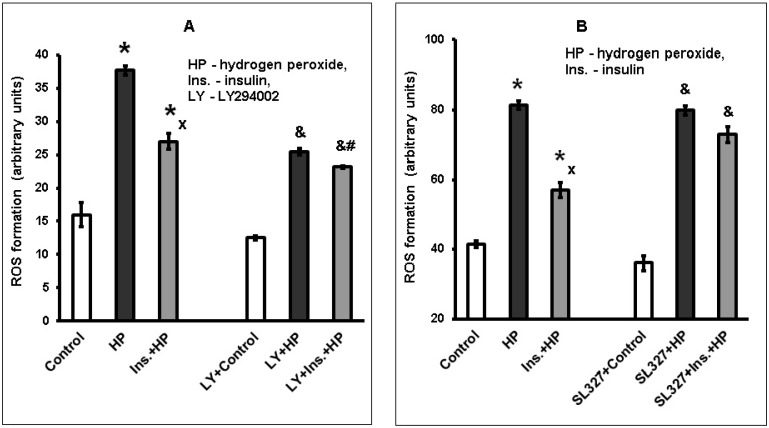
The antioxidant effect of insulin on brain cortical neurons exposed to hydrogen peroxide is diminished in the presence of the inhibitors of LY294002 (**A**) and SL-327 (**B**). The results of one typical experiment (from seven replicates) are given as means ± SEM of four parallel determinations. The differences are significant according to one-way ANOVA as compared: *—to the control values, *p* < 0.01, ^x^—to the effect of hydrogen peroxide alone, *p* < 0.01, ^&^—to the effect of LY29400 and control or SL327 and control, *p* < 0.05, ^#^—to the effect of LY294002 and hydrogen peroxide or SL327 and hydrogen peroxide, *p* < 0.05.

The antioxidant effect of insulin was also shown to decrease in the presence of LY294002 and SL327 (Figure 4A,B). Cortical neurons were incubated with 30 µM LY294002 (A) and with 10 µM SL327 (B) or without them for 30 min. Then neurons were incubated with 100 nM insulin for 1 h and exposed to 100 µM hydrogen peroxide. ROS formation was estimated using dichlorofluorescin diacetate.

One hundred nanomolar insulin inhibited the prooxidant effect of hydrogen peroxide (taken as 100%) by 44.7 ± 8.3% in the absence of the inhibitors, in the presence of 30 µM LY294002 insulin effect was diminished to 22.0 ± 6.1% (*n* = 7), the difference is significant (*p* < 0.05). In another series of experiments, 100 nM insulin inhibited the prooxidant effect of hydrogen peroxide (taken as 100%) by 46.6 ± 10.2% if inhibitors were not used, but in the presence of 10 µM SL327, its inhibitory effect on ROS formation was lower (*p* < 0.05) and constituted 19.2 ± 6.0% (*n* = 5).

### 2.4. Insulin Activates Akt in Brain Cortical Neurons Exposed to Hydrogen Peroxide and in Control Neurons

Akt activity was estimated measuring the level of Akt phosphorylated at Ser^473^, and Akt phosphorylated at Thr^308^ using monoclonal antibodies specific to pAkt (Ser^473^) and pAkt (Thr^308^). It was shown that hydrogen peroxide and insulin are able to increase phosphorylation of both residues. However, the intensity of phosphorylation was much more pronounced at Ser^473^ than at Thr^308^, so the most part of the experiments was done using antibodies against pAkt (Ser^473^). One hundred nanomolar and 1 µM insulin increased the basal Akt activity by 4.8 ± 0.4 and 5.8 ± 0.2 times, respectively (*p* < 0.001 in both cases). 

**Figure 5:**
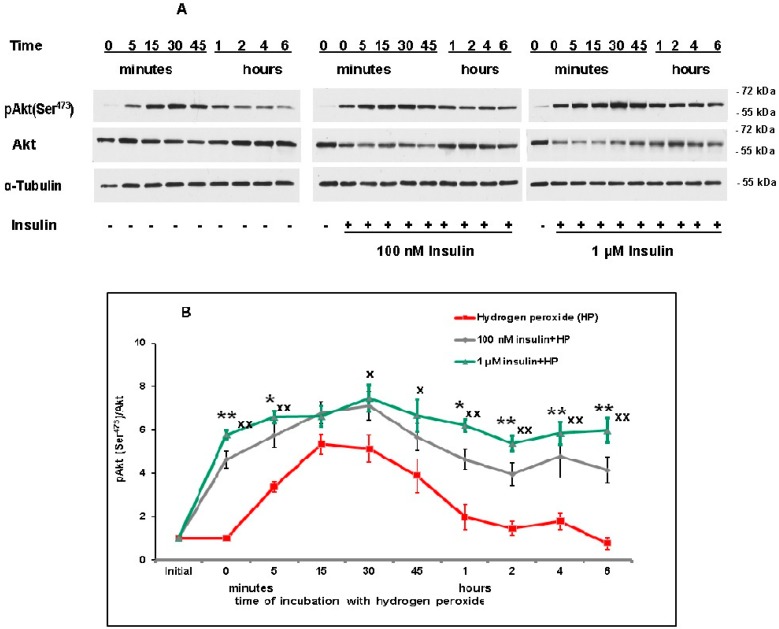
Insulin markedly increases the level of pAkt (Ser^473^) in cortical neurons exposed to hydrogen peroxide. **A**—immunoblots obtained in one typical experiment (from five replicates) are shown. **B**—results of five experiments are presented as means ± SEM. Cortical neurons were incubated with insulin (or without it) for 1 h and then exposed to 100 µM hydrogen peroxide. Red lines with squares show the effect of hydrogen peroxide alone, grey lines with rhombs—the effect of hydrogen peroxide after incubation of neurons with 100 nM insulin, green lines with triangles—the effect of hydrogen peroxide after incubation of neurons with 1 μM insulin. The differences of insulin and hydrogen peroxide effects as compared to the effect of hydrogen peroxide alone on the level of pAkt (Ser^473^) in cortical neurons are significant according to Student’s paired *t* test: * and **—the effect of preincubation with 100 nM insulin is significant, * *p* < 0.05, ** *p* < 0.02, ^x^ and ^xx^—the effect of the preincubation with 1 µM insulin is significant, ^x^ *p* < 0.05, ^xx^ *p* < 0.02.

In our study, it was shown (Figure 5A,B) that hydrogen peroxide itself significantly activated Akt from 5 to 45 min after cortical neuron exposure to this prooxidant, with maximal activation being observed from 15 to 30 min after its application (Figure 5B). It was shown by measuring the ratio pAkt (Ser^473^)/Akt.

At the same time, insulin increased the activity of Akt in cortical neurons (as compared to the effect of hydrogen peroxide alone) at various time of oxidative stress development. Its effect was significant from 5 min to 6 h after the application of prooxidant (for the exception of 15 min interval). It seems of importance that both 100 nM and 1 µM insulin activated Akt 1–6 h after neuron exposure to hydrogen peroxide, when the effect of prooxidant was already absent (Figure 5A,B).

In supplemented materials Figures S2–S5 show how we determined the molecular weights of Akt, GSK-2beta, ERK1/2, and AMPK, respectively, as well as of alpha-tubulin, using protein standards with known molecular weights.

### 2.5. Insulin Increases the Level of pGSK-3beta (Ser^9^) in Brain Cortical Neurons Exposed to Hydrogen Peroxide and Thus Inactivates This Enzyme 

**Figure 6:**
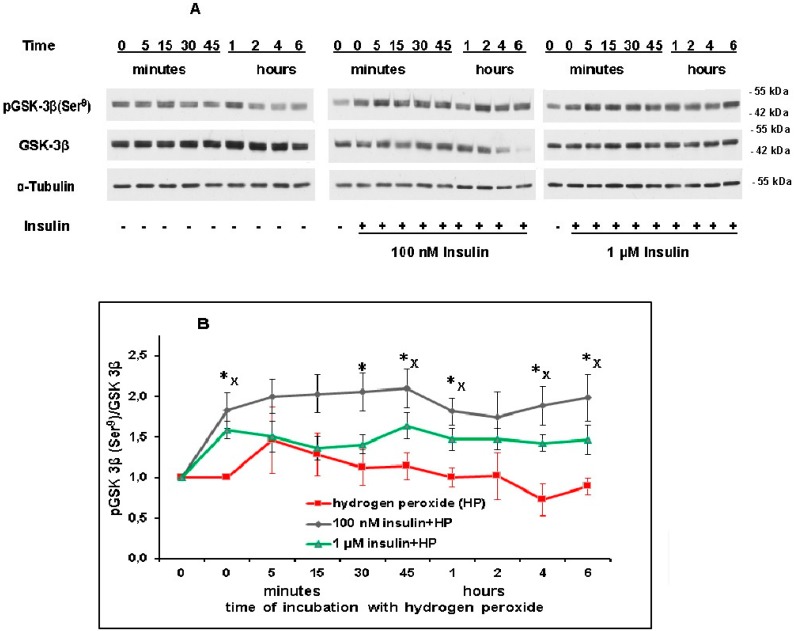
Insulin causes pronounced and significant elevation of the level of pGSK-3beta (Ser^9^) in cortical neurons exposed to hydrogen peroxide. **A**—immunoblots obtained in one typical experiment (from seven replicates) are shown. **B**—results of seven experiments are presented as means ± SEM. Cortical neurons were incubated with insulin (or without it) for 1 h and then exposed to 100 µM hydrogen peroxide. The differences of insulin and hydrogen peroxide effects as compared to the effect of hydrogen peroxide alone on the level of pGSK-3beta (Ser^9^) in cortical neurons are significant according to Student’s paired *t* test: *—the effect of preincubation with 100 nM insulin is significant, *p* < 0.05, ^x^—the effect of the preincubation with 1 µM insulin is significant, *p* < 0.05.

Insulin increases the level of pGSK-3beta (Ser^9^) in brain cortical neurons. This was shown by measuring the pGSK-3beta (Ser^9^)/GSK-3beta ratio. This ratio did not significantly change after neuron exposure to hydrogen peroxide alone. However, after preincubation with 1 µM and 100 nM insulin, the pGSK-3beta (Ser^9^) level increased as compared to its level after exposure to hydrogen peroxide alone at various time points (Figure 6A,B). The preincubation of cortical neurons with 1 µM and 100 nM insulin was found to significantly increase the pGSK-3beta (Ser^9^) level (as compared to its level observed after exposure to hydrogen peroxide alone) 45 min, 1, 4, and 6 h after the beginning of neuron exposure to hydrogen peroxide (Figure 6B). It means that insulin is able to inactivate GSK-3beta in the course of oxidative stress development. In control cortical neurons, 1 µM and 100 nM insulin increased the pGSK-3beta (Ser^9^)/GSK-3beta ratio from 1.0 to 1.8 ± 0.2 and to 1.6 ± 0.1, respectively (*p* < 0.02 in both cases, *n* = 7). 

### 2.6. Insulin Normalizes the Ratio of Pro to Antiapoptotic Proteins (Bax/Bcl-2) in Cortical Neurons Increased by the Cell Exposure to Hydrogen Peroxide 

Pro- and antiapoptotic proteins play important roles in mitochondrial stability and function. In order to prevent mitochondrial destabilization, it is necessary to avoid the elevation of the ratio of pro- to antiapoptotic proteins (Bax/Bcl-2) in various cells including nerve cells.

One hundred nanomolar and 1 µM insulin diminished the basal ratio Bax/Bcl-2 from this value in control neurons, taken as 100% to 74.0 ± 3.1% and 73.5 ± 3.8%, respectively, (*p* < 0.01 in both cases, *n* = 9). Insulin appears to have favorable effect on control neurons.

**Figure 7:**
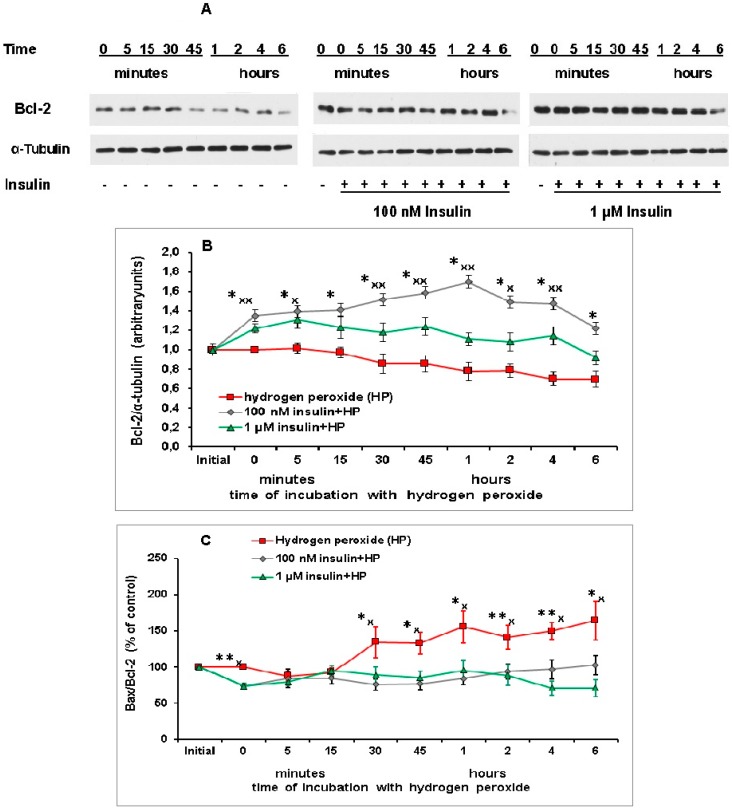
Insulin normalizes the Bax/Bcl-2 ratio in cortical neurons markedly elevated by their exposure to hydrogen peroxide. Cortical neurons were incubated with insulin for 1 h (or without it) and then exposed to hydrogen peroxide. **A**—immunoblots showing the effect of insulin and hydrogen peroxide on Bcl-2 level are shown, they were obtained in one typical experiment (from nine replicates). In **B** (the level of Bcl-2) and **C** (Bax/Bcl-2 ratio) the data are presented as means ± SEM from nine experiments made. The differences of insulin and hydrogen peroxide effects as compared to the effect of hydrogen peroxide alone on the Bcl-2 level (**B**) and on Bax/Bcl-2 ratio (**C**) in cortical neurons are significant according to Student’s paired *t* test. **B**. *—the effect of preincubation with 100 nM insulin is significant, *p* < 0.02, ^x^ и ^xx^—the effect of preincubation with 1 µM insulin is significant, ^x^ *p* < 0.05, ^xx^ *p* < 0.02. **C**. * and **—the effect of preincubation 100 nM insulin on Bax/Bcl-2 ratio in cortical neurons is significant, * *p* < 0.05, ** *p* < 0.01, ^x^—the effect of preincubation with 1 µM insulin on Bax/Bcl-2 ratio in neurons is significant, *p* < 0.02.

It was most important for us to study the effect of insulin on Bax/Bcl-2 ratio in brain cortical neurons exposed to prooxidant. The level of Bcl-2 was found to decrease gradually in these neurons after application of hydrogen peroxide (Figure 7A,B). This diminution reached 20–30% of control level 1–6 h after the beginning of prooxidant action. Preincubation with 100 nM and 1 µM insulin significantly increased the Bcl-2 level in neurons at various times after the exposure to hydrogen peroxide (Figure 7B). 

In supplemental materials Figure S6 shows how we determined the molecular weight of Bcl-2 and of alpha-tubulin using protein standards with known molecular weights. 

The ratio of pro- to antiapoptotic protein Bax/Bcl-2 (Figure 7C) is significantly increased by 41–64% in brain cortical neurons at various times after the neuron exposure to hydrogen peroxide (30 and 45 min, 1, 2, 4 and 6 h). Preincubation with 100 nM and 1 µM insulin prevented this effect of prooxidant and normalized this ratio in brain cortical neurons, Bax/Bcl-2 ratio became much lower than at the action of hydrogen peroxide alone and practically similar to this ratio in control cells (Figure 7C). 

### 2.7. Insulin and Hydrogen Peroxide Activate ERK1/2 in Cortical Neurons 

**Figure 8:**
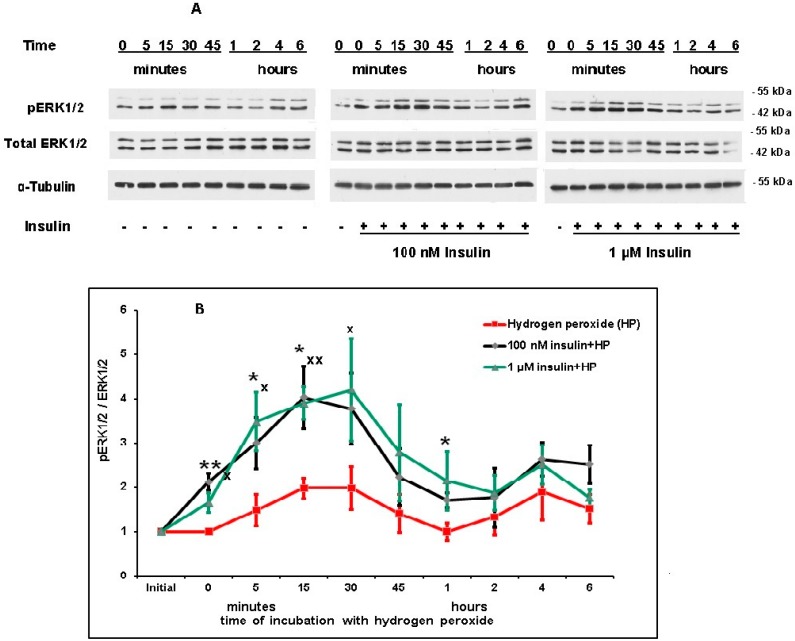
Insulin increases the level of pERK1/2 in cortical neurons at early stages of cell exposure to hydrogen peroxide. **A**—immunoblots obtained in one typical experiment (from five replicates) are shown. **B**—results of five experiments are presented as means ± SEM. Cortical neurons were preincubated with 1 µM or 100 nM insulin (or without it) for 1 h and then exposed to 100 µM hydrogen peroxide. The differences of insulin and hydrogen peroxide effects as compared to the effect of hydrogen peroxide alone on the level of pERK1/2 (pERK1/2/ERK1/2 ratio) in cortical neurons are significant according to Student’s paired *t* test: * and **—the effect of preincubation with 100 nM insulin is significant, * *p* < 0.05, ** *p* < 0.01, ^x^—the effect of preincubation with 1 µM insulin is significant, *p* < 0.05.

The ERK1/2 activity was estimated by the determination of the level of phosphorylated form of this enzyme using a monoclonal antibody specific to pERK1 (pThr^202^/pTyr^204^) and pERK2 (pThr^185^/pTyr^187^). It was shown that hydrogen peroxide activated this protein kinase, significantly increasing the level of its phosphorylated form 15 min after its application (Figure 8A,B). Preincubation of neurons with 100 nM and 1 µM insulin significantly enhanced ERK1/2 activity (the level of pERK1/2) at early stages of hydrogen peroxide action (5 and 15 min after the prooxidant application). Besides, 1 µM insulin activated ERK1/2 30 min after neuron exposure to prooxidant and 100 nM insulin—1 h after prooxidant application (Figure 8B). Preincubation with insulin had no effect on ERK1/2 activity at later periods of oxidative stress development (2–6 h after application of prooxidant).

In order to evaluate the changes in the expression of Akt, GSK-3beta, and ERK1/2, their level was normalized for alpha-tubulin. The ratio of each of these protein kinases to alpha-tubulin was not altered as a result of cortical neuron exposure to hydrogen peroxide or insulin [22]. It means that Akt, GSK-3beta, and ERK1/2 expression does not significantly change in the course of oxidative stress development. 

### 2.8. Insulin Inhibits the Activity of AMPK in Cortical Neurons Activated by Exposure to Hydrogen Peroxide 

**Figure 9:**
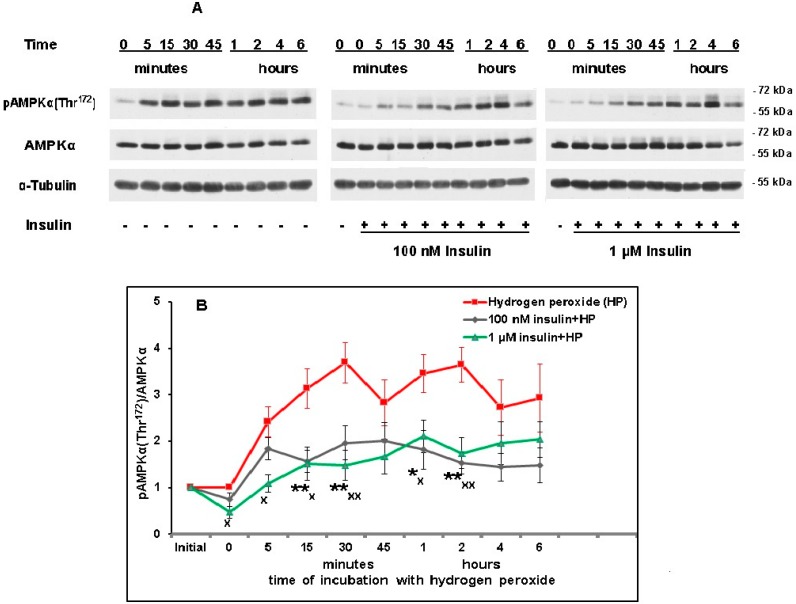
Insulin significantly diminishes the level of pAMPKalpha (Thr^172^) markedly elevated in cortical neurons by their exposure to hydrogen peroxide. **A**—immunoblots obtained in one typical experiment (from seven replicates) are shown. **B**—results of seven experiments are presented as means ± SEM. Cortical neurons were incubated with insulin (or without it) for 1 h and then exposed to 100 µM hydrogen peroxide. The differences of insulin and hydrogen peroxide effects as compared to the effect of hydrogen peroxide alone on the level of pAMPKalpha (Thr^172^) in cortical neurons are significant according to Student’s paired *t* test: * and **—the effect of preincubation with 100 nM insulin is significant, * *p* < 0.05, ** *p* < 0.02, ^x^ and ^xx^—the effect of preincubation with 1 µM insulin is significant, ^x^ *p* < 0.05, ^xx^ *p* < 0.02.

Hydrogen peroxide was found to activate AMPK activity in cortical neurons at various time intervals after the cell’s exposure to this prooxidant, increasing the degree of its phosphorylation at Thr^172^ (Figure 9A,B). The activation of this enzyme is characteristic of various stresses, including oxidative stress. The difference between AMPK activity in control neurons taken as 1.0 and that in neurons exposed to hydrogen peroxide for 15, 30, 45 min and for 1, 2, 4, and 6 h was significant according to one-way ANOVA followed by Dunnetts’s test for multiple comparisons (*p* < 0.05 in all cases). Insulin inhibited the activity of this enzyme at the time of its most pronounced activation by hydrogen peroxide as one can see from the data presented in Figure 9B, preincubation with 100 nM and 1 µM insulin prior to prooxidant exposure significantly decreased AMPK activity 15, 30 min, 1, and 2 h after neuron exposure to hydrogen peroxide.

In order to evaluate the changes in the expression of AMPK its level was normalized for alpha-tubulin. The ratio AMPK/alpha-tubulin was not altered after cortical neuron exposure to hydrogen peroxide or insulin [22]. It means that AMPK expression does not significantly change in the course of oxidative stress development. 

In the presence of the AMPK activator (AICAR), the neuroprotective effect of insulin significantly diminished. Its rescue rates fell from 38.6 ± 5.3 % in the absence of modulators to 19.0 ± 5.5% in the presence of 100 µM of AICAR (*p* < 0.05). Thus, the insulin-induced inhibition of AMPK activity in cortical neurons in oxidative stress appears to contribute to its protective effect.

In order to evaluate the changes in the expression of AMPK its level was normalized for alpha tubulin, this ratio was not changed as a result of cortical neuron exposure to hydrogen peroxide or insulin [22]. 

### 2.9. Insulin Increases the Mitochondrial Membrane Potential (∆ψ(m)) in Cortical Neurons Diminished after Cell Exposure to Hydrogen Peroxide 

**Figure 10:**
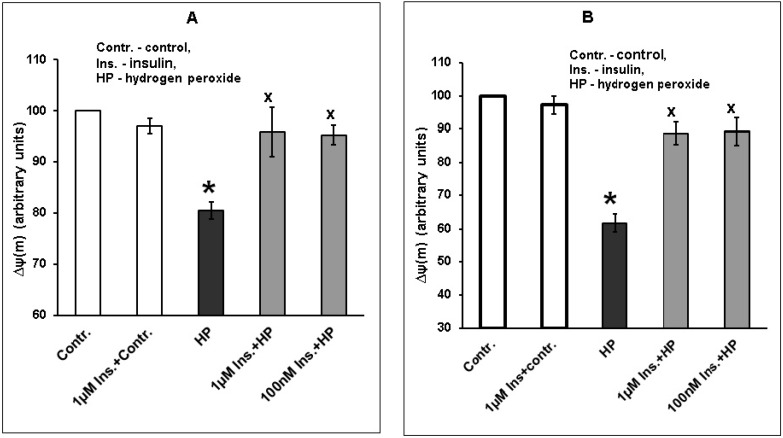
Insulin diminishes the drop in mitochondrial membrane potential in cortical neurons caused by their exposure to hydrogen peroxide. The results of one typical experiment (from five replicates) are given as means ± SEM of four parallel determinations. Cortical neurons were incubated with 1 µM and 100 nM insulin: **A**—for 1 h, **B**—for 20 h. Then neurons were exposed to 100 µM hydrogen peroxide. The differences are significant according to one-way ANOVA as compared: *—to control values, *p* < 0.02, ^x^—to the effect of hydrogen peroxide alone, *p* < 0.05.

We studied the effect of insulin on ∆ψ(m) in cortical neurons under conditions of oxidative stress. Exposure to hydrogen peroxide was found to cause a drop of ∆ψ(m) in cortical neurons, at the same time, preincubation with 1 µM and 100 nM insulin for 1 or 20 h partially or completely normalized this parameter, significantly increasing it (Figure 10A,B). 

In order to take into account all experiments performed, we calculated the diminution of hydrogen peroxide effect on ∆ψ(m) caused by neuron exposure to insulin. The value of ∆ψ(m) decrease caused by exposure to hydrogen peroxide was taken as 100%. Preincubation of cortical neurons with 1 µM and 100 nM insulin for 1 h (*n* = 5) reduced this decrease of ∆ψ(m) by 83.8 ± 10.5% and 69.6 ± 14.9%, respectively, while preincubation for 20 h (*n* = 5) reduced it by 69.5 ± 7.9% and 61.1 ± 10.4%, respectively. 

**Figure 11:**
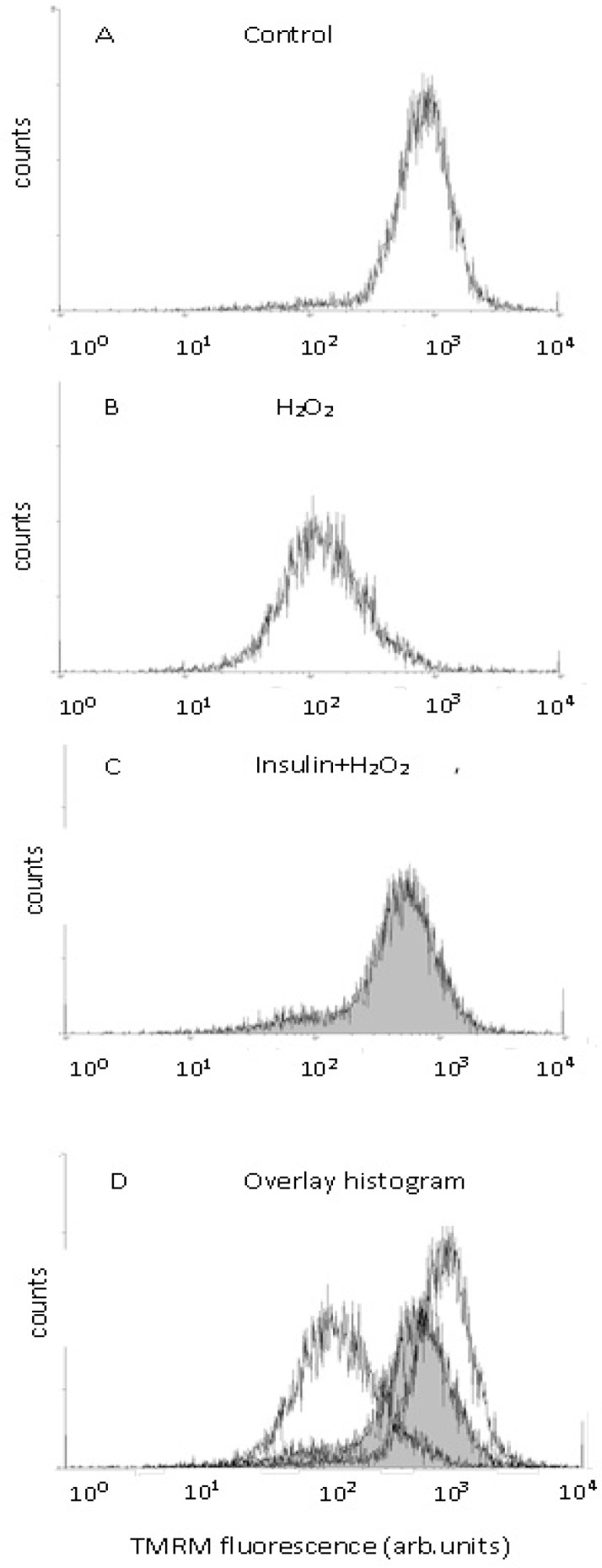
Effect of hydrogen peroxide and insulin on the mitochondrial membrane potential in cortical neurons (histograms obtained by flow cytometry). Cortical neurons were incubated with 1 µM insulin for 1 h, then neurons were exposed to 100 µM hydrogen peroxide. **A**—control, **B**—the effect of hydrogen peroxide alone, **C**—the effect of hydrogen peroxide after cell preincubation with 1 µM insulin, **D**—the overlay of the histograms presented in **A**–**C**.

The results of a typical experiment are presented as histograms obtained from a flow cytometer in Figure 11. After neuron exposure to hydrogen peroxide, the main peak moves left to the region where cells with a smaller ∆ψ(m) value are present. After preincubation with 1 µM insulin, the main peak is found closer to the control peak than in the case of exposure to the prooxidant alone. Thus, preincubation with insulin diminishes, to a large extent, the hydrogen-peroxide-induced decrease of the mitochondrial membrane potential in cortical neurons.

## 3. Discussion

Insulin’s functions in the central nervous system and in extraneural organs are quite different. Insulin regulates peripheral glucose uptake and glucose production within the liver. The main regulatory mechanism by which glucose uptake takes place is via insulin-stimulated transport of glucose into skeletal muscle and adipose tissue, primarily mediated by glucose transporter protein type-4 (GLUT4). GLUT4 is a key component in glucose homeostasis and the removal of glucose from circulation [23,24,25]. However, GLUT4 is absent in the most part of brain regions, at the same time it is a minor component of glucose transporters in some brain regions. The main glucose transporters found in brain tissue are GLUT1 (characteristic for endothelial and astroglial cells) and GLUT3 (characteristic for neurons). Both GLUT1 and GLUT3 appear to be insulin-insensitive [23,24,25]. That is why for a long time it was believed that brain is insulin-insensitive organ and its function in brain was studied less than in peripheral organs and tissues. In recent years, the interest in insulin function in the central nervous system is very high. Insulin’s action on hypothalamic neurons was shown to regulate energetic metabolism and the function of endocrine systems due to the production of hypothalamic releasing factors by these neurons [20,26]. Administration of insulin to animals or humans suffering from neurodegenerative or diabetic diseases was shown to ameliorate the cognitive functions and to increase the viability of brain neurons [4,5,6,27,28].

The insulin doses administered to humans intranasally in order to improve their cognitive functions are usually 20 or 40 IU, 40 IU was shown to have more pronounced positive effect on people with MCI or Alzheimer’s disease, see, for example, [6,28]. Intranasally administered insulin was shown to reach brain directly bypassing blood–brain barrier [29]. One IU of insulin is equal to 0.0347 mg of human insulin or approximately to 7 nmoles of this hormone. Forty IU corresponds to 280 nmoles of insulin. If 10% of intranasally administered insulin reaches human cerebrospinal fluid (CSF), exogenous insulin concentration in human CSF (volume 140–270 mL) may be anticipated to be approximately 140 nM. Though there are many assumptions in these arguments, exogenous insulin concentrations which improve brain function in clinical trials appear to be comparable with insulin concentrations which increase the viability of cultured cortical neurons in vitro (in our experiments maximal insulin effect was observed at the action of 100 nM insulin).

The insulin-induced increase of viability of cultured neurons and neuronal cell lines exposed to prooxidants and other toxins has been demonstrated in a large number of studies, see, for example [11,17,30]. However, the mechanism of neuroprotective insulin action is far from being understood. 

Activation of Akt plays the most important role in insulin protection [8,9,10,11,12]. It may lead to phosphorylation and inactivation of GSK-3beta, which has regulatory effects on mitochondrial biogenesis, bioenergetics, permeability, and apoptosis. Activation of GSK-3beta results in the drop of [∆ψ(m)], opening of mPTP, and apoptotic death of various cells, while its inactivation prevents the death of the cells [16,31,32]. Besides, Akt activation and downstream activation of the CREB transcription factor may lead to the increased expression of Bcl-2 and to the decrease of pro- to antiapoptotic proteins ratio [13,14]. Anti- and proapoptotic proteins of Bcl-2 family are important regulators of apoptosis. If the ratio of mitochondrial pro- to antiapoptotic proteins is high, mitochondria initiate programmed cell death, releasing AIF, cytochrome c, and other proapoptotic factors. 

Our data on insulin-induced protein kinase B (Akt) activation in cortical neurons are in agreement with the data of other authors showing that insulin activates this protein kinase in nerve and other cells [8,9,12,17,33,34]. Hydrogen peroxide itself activates Akt. Insulin increases the activity of Akt in cortical neurons (as compared to the effect of hydrogen peroxide alone) from 5 min to 6 h after application of prooxidant (for the exception of 15 min interval). It seems of importance that 100 nM and 1 µM insulin is able to activate Akt 1–6 h after neuron exposure to hydrogen peroxide, when the activating effect of prooxidant is already absent (Figure 5A,B). These results are in agreement with the data of Huang and coauthors, who found that insulin is able to activate Akt in adult sensory neurons both rapidly, and for many hours, improving the mitochondrial function [33].

As far as the enzymes downstream of Akt are concerned, we have shown that preincubation of cortical neurons with 100 nM and 1 µM insulin results in a pronounced and significant rise in pGSK-3beta (Ser^9^) level in neurons and thus in inactivation of this enzyme 45 min, 1, 2, 4, and 6 h after the cell’s exposure to hydrogen peroxide. Our results do not agree with the data of Duarte and coauthors [17]. They found that preincubation of cortical neurons with 100 nM and 10 µM insulin did not change the level of pGSK-3beta (Ser^9^) 5 h after the cell’s exposure to prooxidants. These authors suggest that insulin-induced inactivation of pGSK-3beta results from the diminution in the level of pGSK-3beta (Tyr^216^) in neurons instead of a rise in a phosphorylation of the enzyme at Ser^9^ [17]. The phosphorylation of GSK-3beta at Tyr^216^ was previously described and suggested to be a result of enzyme autophosphorylation [35]. According to our results, insulin had no effect on pGSK-3beta (Tyr^216^) level in cortical neurons. At the same time, our results are in agreement with the data showing the insulin-induced elevation of pGSK-3beta (Ser^9^) level in hippocampi of ischemic and diabetic mice [36]. They are consistent with the data of Kim and coauthors [21], as the insulin-induced rise of basal pGSK-3beta (Ser^9^) level in cortical neurons was shown both by these authors and by us (see Results). It should be taken into account that the IGF-1-induced increase of pGSK-3beta (Ser^9^) is characteristic for cerebellar granule neurons and catecholaminergic neurons [15,37]. Thus, it appears that the main way of GSK-3beta inactivation characteristic for insulin-induced protection of various nerve cells, including cortical neurons, is enzyme phosphorylation at Ser^9^. 

It is of interest that the mechanism of action of GSK-3beta activation on mitochondrial dysregulation is rather complicated. It includes the GSK-3beta-dependent phosphorylation of Bax at Ser^163^ that facilitates its translocation to mitochondria and the effect of GSK-3beta on the voltage-dependent anion and ATP-dependent potassium channels and on cyclophylin D and ANT interaction [16]. 

Our results evidencing the insulin-induced increase of Bcl-2 level (Figure 7A,B) are in agreement with previously obtained data [17]. According to our data, hydrogen peroxide causes a significant increase of the Bax/Bcl-2 ratio in brain cortical neurons 1–6 h after the beginning of neuron exposure to this prooxidant. At the same time preincubation with insulin normalizes this ratio in the neurons under conditions of oxidative stress and makes it similar to that in the control cells (Figure 7C). 

We have found that the exposure of cortical neurons to hydrogen peroxide leads to a marked increase of ROS formation and to a drop of mitochondrial inner membrane potential in these cells, while preincubation of neurons with 100 nM and 1 µM insulin results in a significant diminution of ROS formation and in a normalization of ∆ψ(m) in neurons. 

The data obtained by us are in agreement with previously obtained results of other authors. Insulin was shown to diminish significantly the ROS formation in cultured nerve cells and neuronal cell lines exposed to various prooxidants and to increase viability of these cells [11,30,38,39]. The antioxidant effect of insulin was also demonstrated in vivo, as insulin diminished ROS formation and lipid peroxidation product accumulation in brain that is characteristic for many diseases [38,39,40,41]. Thus, our results showing that insulin diminishes the hydrogen peroxide-induced ROS formation in nerve cells confirms the data previously obtained by other authors on the antioxidant properties of insulin.

It is of interest that insulin, after its application to the cells, increases at first to a certain extent the ROS formation (by means of NOX4 activation) and thus triggers various protein kinase activation, in particular activation of Akt and ERK1/2 [42,43]. But, later on, the insulin-induced modulation of protein kinase activities leads to decrease of ROS formation in the cells. 

The data showing insulin’s effect on mitochondrial membrane potential in nerve cells are less abundant. Our data are in agreement with the data of Song and coauthor [39]. In this in vivo study, a piece of work was performed on the cells of neuronal cell line PC12. It was shown that exposure to hydrogen peroxide greatly diminished the value of mitochondrial membrane potential in these cells, but preincubation with insulin significantly and markedly increased it.

It may be assumed that the insulin-induced inactivation of GSK-3beta and the decrease of Bax/Bcl-2 ratio in cortical neurons exposed to prooxidant are of importance for its ability to diminish the ROS formation and to normalize the mitochondrial membrane potential in these cells (or decrease a drop in its level). Thus, there is evidence that the elevation of Bcl-2 level has a pronounced antioxidant effect in various cells [44,45,46]. Bax, on the contrary, was found to initiate ROS production in the cells, except when coexpression of Bcl-xL and Bax prevented this effect [44]. Oxidative stress was shown to lead to a fall of ∆ψ(m) and to the opening of mPTP in cells. mPTP opening, in its turn, was found to lead to further activation of free radical reactions in cells [47,48]. Cell exposure to prooxidants results in a fall of ∆ψ(m), while the phosphorylation of GSK-3beta at Ser^9^ and its inactivation induced by protectors diminishes ROS formation and a drop in ∆ψ(m) in the cells [16,31,32]. It is only a suggestion that insulin-induced inactivation of GSK-3beta due to its phosphorylation at Ser^9^ and the normalization of Bax/Bcl-2 ratio in cortical neurons make pronounced contribution to its antioxidant effect in our experiments. Further studies are needed to make a conclusion that protective effect of insulin depends to a great extent on its ability to diminish ROS formation in nerve cells.

Thus, insulin-induced activation of Akt and downstream inactivation of GSK 3-beta as a result of its phosphorylation at Ser^9^ and the diminution of Bax/Bcl-2 ratio appear to make a pronounced contribution to protective effect of insulin on brain cortical neurons under conditions of oxidative stress. This conclusion is confirmed by our data on the pronounced and significant lowering of the protective and antioxidant effects of insulin on cortical neurons under conditions of oxidative stress in the presence of the PI3K/Akt pathway inhibitor LY294002 (see Figure 3A and Figure 4A and, Table 1). It should be noted that LY294002 diminished, but did not abolish, the protective effect of insulin. The rescue rate of insulin in the presence of this inhibitor (20.9 ± 6.3%) is a significant value. It suggests the contribution of other signaling pathways that also determine the ability of insulin to enhance the neuronal viability. 

The activation of ERK1/2 may be one of such insulin effects. The data showing that insulin activates ERK1/2 in the cells of extraneural organs are numerous. However, the data on insulin modulation of ERK1/2 activity in neurons are contradictory. Thus, data were obtained suggesting that insulin had no effect on the activity of ERK1/2 (the level of pERK1/2) in brain cortical neurons [17] and that the protective effect of insulin did not depend on the activation of ERK1/2 in these neurons [49].

According to our data, the level of pERK1/2 was significantly higher in samples preincubated with 100 nM or 1 µM insulin prior to hydrogen peroxide application than in samples which were incubated with hydrogen peroxide alone, if determinations were made 5 or 15 min after prooxidant application. Besides, 1 µM insulin significantly increased ERK1/2 activity 30 min after neuron exposure to prooxidant and 100 nM insulin 1 h after its application (Figure 8). Thus, according to our data, insulin activated ERK1/2 in cortical neurons at early stages of oxidative stress development but it had no effect on ERK1/2 activity 2–6 h after application of prooxidant. It may be noted that the exposure of Chinese hamster ovary cells overexpressing insulin receptors to insulin resulted in transient activation of ERK1/2 peaked at about 5 min, then the activity declined rapidly to about baseline within 30 min [50]. At the same time, the absence of an effect of insulin on pERK1/2 level in cortical neurons was demonstrated measuring the level of pERK1/2 at late period of oxidative stress development 5 h after the application of prooxidants [17]. Most probably, it was too late to reveal the activating effect of insulin on ERK1/2 at that time. IGF-1 was shown to activate ERK1/2 in small trigeminal ganglion neurons [51] and, in R28 cells, differentiated to model retina neurons [52]. It was also shown that insulin activates ERK1/2 signaling in the dorsal vagal complex [53]. 

According to our data, the inhibitor of the MEK1/2/ERK1/2 (SL327) signaling pathway significantly decreases the rescue rates of insulin in cortical neurons under conditions of oxidative stress from 41.65 ± 2.4% to 19.9 ± 5.6% (Table 1), see also the results of a typical experiment (Figure 3B). This inhibitor was shown to diminish the antioxidant effect of insulin as well (Figure 4B). Thus, the insulin-induced activation of ERK1/2 at early stages of oxidative stress development in cortical neurons appears to contribute to the ability of insulin to promote neuronal viability and inhibit the oxidative stress development in the cells. 

It is of interest that short activation of ERK1/2 by neurotrophins, flavonoids, gangliosides, and other compounds increases the viability of nerve cells [54,55]. According to our data insulin appears to belong to such protectors. But long persistent activation of ERK1/2 caused by ischemic or other brain injury, on the contrary, leads to neuronal death, the inhibitors of ERK1/2 diminish the death of the brain neurons and improve the general state of ischemic and reperfused animals (see, for example, [56]). Alpha-tocopherol [57,58] and carnosine [59] prevent or diminish the long or persistent activation of ERK1/2 in cultured nerve cells, inhibiting this enzyme at the late stages of oxidative stress development, it leads to elevation of their protective effect.

We could not find published data showing how insulin modulates AMPK activity in nerve cells with the exception of various hypothalamic regions [20]. According to our data, hydrogen peroxide activated AMPK in cultured cortical neurons at various time after neuron exposure to this prooxidant. The activation of AMPK is characteristic for stresses, including oxidative stress. It is concerned with ATP depletion and accumulation of AMP and ADP in various cells [60]. Preincubation of neurons with 100 nM and 1 µM insulin significantly diminished AMPK activity at the time of its most pronounced activation by hydrogen peroxide (Figure 9A,B). Our results are in agreement with the data on insulin-induced inhibition of AMPK in various hypothalamic regions [20] and in extraneural organs and cells. Thus, insulin was shown to decrease AMPK activity in hepatocytes, myotubes, skeletal muscle, and heart [61,62]. It was also found to suppress activation of AMPK in cultured adipocytes [63]. The insulin-induced inhibition of AMPK promotes its anabolic effects [61]. 

AMPK plays a dual role in neuroprotection and neurodegeneration. Its activation plays an important role in the protective effect of a large number of compounds. For example, activation of AMPK by metformin and other substances diminishing cell resistance to insulin plays an important role in their protective effects [64]. On the other hand, the excessive activation of AMPK may be responsible for cell death. Thus, hyperactive AMPK appears to play an important role in Alzheimer’s disease pathophysiology [65]. Activation of AMPK in cultured hippocampal neurons was shown to mediate the synaptotoxic effects of amyloid beta-peptide oligomers, it appears to play an important role in the development of Alzheimer’s disease at its early stages [66]. Pathological activation of AMPK-alpha1 is characteristic of murine neonatal hypoxia-ischemia and of oxygen/glucose deprivation in neurons [67]. We found (see Results) that in the presence of the AMPK activator (AICAR), the neuroprotective effect of insulin is significantly diminished (*p* < 0.05). Thus, insulin inhibits the activity of AMPK which is activated in nerve cells under conditions of oxidative stress, the data obtained suggest that this effect of insulin contributes to its protective effect.

In recent years, the administration of intranasal insulin and of drugs which enhance cell sensitivity to this hormone was shown to improve cognitive function in patients with Alzheimer’s disease, mild cognitive impairments, or diabetes mellitus, as well as in animal models of these diseases [4,5,6,27,28,30,68]. It was shown that intranasal insulin has direct impact on brain [29]. At the same time, long administration of high doses of insulin, especially when it is used as a monotherapy, may result in the development of cell resistance to this compound and in the decrease of neuronal viability [4,21,69,70]. That is why the studies of the mechanisms of protective action of insulin and other compounds that inhibit free radical reactions and enhance neuronal viability (and the comparison of their effects on signaling systems) are of importance for targeted search of the protectors that are able to increase (additively or synergistically) the neuroprotection by insulin. The common use of insulin and such protectors (if they were revealed) may lead to the diminution of insulin doses used and to an increase of its effectiveness.

## 4. Materials and Methods

### 4.1. Materials

Hydrogen peroxide, NADH, insulin, poly-D-lysine, sodium dodecyl sulfate, 3-(4,5-dimethylthiazol-2-yl)-2,5-diphenyltetrazolium bromide (MTT), dichlorofluorescin diacetate, AICAR and BMS-754807 were purchased from Sigma-Aldrich (Saint-Louis, Missouri, USA), LY294002 and SL327 were from Tocris (Ellisville, Missouri, USA). The Neurobasal medium, B27 Supplement, B27 Supplement without insulin, penicillin/streptomycin solution and Glutamax were purchased from Gibco (Paisley, UK). Hanks buffered salt solution and trypsin:versen (1:1) solution were from the Biolot Company (Saint-Petersburg, Russia), dimethylformamide was bought from Vecton company (Saint-Petersburg, Russia). The antibodies and other reagents used for immunoblotting are listed in the corresponding Section 4.6. 

### 4.2. Brain Cortical Neurons in Culture

Primary cultures of immature brain cortical neurons were prepared from embryonic day 17–18 Wistar rat fetuses as previously described [71]. All procedures using animals were in accordance with the European Community Council Directive 1986 (2010/63/EEC) and “Guide for the care and use of laboratory animals”, they were approved by the Ethical animal care and use committee of the Institute of evolutionary physiology and biochemistry of Russian Ac. Sci. The cells were seeded on poly-D-lysine-coated 24-well and 12-well plates at a density of 5 × 10^5^ and 1 × 10^6^ cells per well, respectively, in Neurobasal medium containing 2% of B27 Supplement, 2 mM Glutamax, 100 U/mL of penicillin and 100 µg/mL of streptomycin (growth medium). Half of the culture medium was replaced every three days or every second day. The experiments started on the 5th–6th day in vitro of cell cultivation in the growth medium. The growth medium was changed to Neurobasal medium containing 1% B27 without insulin, the neurons were left in it over night before the start of the experiments.

### 4.3. Determination of the Viability of Brain Cortical Neurons, Caspase-3 Activity, and of the Level of Cleaved Caspase-3

Preincubation of neurons with insulin at various concentrations was performed in Neurobasal medium containing 1% B27 without insulin for 1 h. Then the cells were exposed to 50 µM or 100 µM hydrogen peroxide for 6 h. If necessary, neurons were preincubated with 30 µM LY294002, 10 µM SL327, 1 µM BMS-754807, or 100 µM AICAR for 30 min prior to application of insulin. To evaluate the viability of neurons colorimetric MTT method was used, which is based on the reduction of 3-(4,5-dimethylthiazol-2-yl)-2,5-diphenyltetrazolium bromide (MTT) to purple-colored MTT-formazan by the mitochondria of viable cells. MTT reduction assay was performed as previously described [72]. The absorbance of MTT-formazan was measured at 575 nm in a microplate reader. Extinction of control samples was taken as 100%, the extinction of other samples was expressed as percent of control values. 

Caspase activity in neuronal cell cultures was determined using the caspase-3 assay kit (CASP3F-1KT, Sigma-Aldrich, USA), following the manufacturer’s instructions. Fluorescence was measured using plate reader Fluoroscan Ascent Fl (Thermo Fischer Scientific, Vantaa, Finland).

### 4.4. Determination of ROS Formation In Brain Cortical Neurons

Neurons were incubated with 1 µM or 100 nM insulin (or without it) for 1, 5, or 20 h in Neurobasal medium containing 1% of B27 Supplement without insulin. After removing the medium, the fluorescent dye dichlorofluorescin diacetate was added to the brain cortical neurons in Hanks’ balanced salt solution (HBSS) to a final concentration of 10 µM. The neurons were incubated with the dye for 40 min in the dark at 37 °C, washed twice with HBSS, and exposed to 100 µM hydrogen peroxide for 1 h. The fluorescence of the reaction product of dichlorofluorescin with ROS was determined using a Fluoroscan Ascent FL (Thermo Fisher Scientific, Finland) fluorometer for plates (excitation at λ = 485 nm, emission measured at λ = 538 nm). Arbitrary units were used to determine the ROS level. 

### 4.5. Evaluation of Insulin and Hydrogen Peroxide Effects on the Mitochondrial Membrane Potentials (∆ψ (m)) in Brain Cortical Neurons

Determination of ∆ψ (m) in brain cortical neurons was performed by the flow cytometry method using the fluorescent dye tetramethylrhodamine (TMRM) [73]. The growth medium was changed to Neurobasal medium 18 h prior to the beginning of the experiments. Preincubation of cortical neurons with insulin took place for 1 or 20 h in this medium, afterward the neurons were exposed to 100 µM hydrogen peroxide for 1 h. The cells were taken off the wells by trypsin–versen (1:1) and washed by HBSS. The residue was resuspended in HBSS containing 100 nM TMRM. Then it was incubated for 30–40 min at 37 °C in the dark. The samples were analyzed by flow-cytometry method using cytometer Beckman Coulter Epics XL, USA. Fluorescence was measured at 575 nm in an F2 channel. In each sample, 10,000 events were recorded. The intensity of fluorescence was shown on logarithmic scale, it was expressed in arbitrary units as the mean intensity of fluorescence. Cytofluorometric data were analyzed using the program WinMDI, version 2.9. Fluorescence of various samples was expressed as percent of fluorescence, with control cells taken as 100%.

### 4.6. Evaluation of Insulin and Hydrogen Peroxide Effects on Akt, GSK-3beta, ERK1/2, and AMPK Activities and Expression of These Protein Kinases, Bax, Bcl-2, and Cleaved Caspase-3 Using Western Blot Analysis

After incubation with insulin (or without it) for 1 h and with 100 µM hydrogen peroxide for 6 h, brain cortical neurons were washed twice with ice-cold phosphate buffer solution (PBS) and harvested in 60 µL of lysis buffer. The lysis of cortical neurons and determination of protein concentrations were performed as previously described [58]. Equivalent amounts of lysates (20–25 µg of protein) were loaded into each lane, they were subjected to electrophoresis in 10% sodium dodecyl sulfate polyacrylamide gel. Afterwards, their transfer to 0.45 µm Protran nitrocellulose membranes (Amersham, GE Healthcare, Buckinghamshire, UK) took place. The nonspecific binding sites of the membranes were blocked as previously described [58]. The blots were then probed overnight with monoclonal antibodies specific for pAkt (Ser^473^) (#4058) and pAkt (Thr^308^) (13038) (1:1000, Cell Signaling Technology, Beverly, Massachusetts, USA)., for pGSK-3beta (Ser^9^) (1:1000, #9322, Cell Signaling Technology, USA), for pERK1 (pThr^202^/pTyr^204^) and pERK2 (pThr^185^/pTyr^187^) (1:2000, #E7028, Sigma-Aldrich, USA), for pAMPK-alpha (Thr^172^) (#2535) and for cleaved caspase-3 (Asp175) (#9664) (1:1000, Cell Signaling Technology, USA). The specific antibodies to total ERK1/2 (#9102), Akt (#4691), GSK-3beta (#9315) and AMPK-alpha (#2532) (1:1000, Cell Signaling Technology, USA) were used to determine the level of these enzymes and the possible changes in their expression. The level of Bcl-2 (#2870) and Bax (#2772) in neurons was determined using antibodies specific to them (1:1000, Cell Signaling Technology USA). The blots were washed three times with 0.1% Tween 20 in Tris-buffered saline and incubated with either an anti-mouse (#7076) or anti-rabbit (#7074) HRP-IgG secondary antibody (Cell Signaling Technology, USA) for 1 h at room temperature. The ratios pERK1/2/ERK1/2, pAkt (Ser^473^)/Akt, pAkt (Thr^308^)/Akt, pGSK-3beta (Ser^9^)/GSK-3beta, pAMPK-alpha (Thr^172^)/AMPK-alpha were determined and taken as 1.0 in control cells. In order to normalize the data, membranes after stripping were stained for alpha-tubulin (1:2000, #T6074 Sigma-Aldrich, USA). To check the changes of the expression of total ERK1/2, Akt, GSK-3beta, and AMPK, their ratio to alpha-tubulin was determined and taken as 1.0 in control neurons. The stripping procedure was previously described [58]. The membranes were re-probed with antibodies for total above-mentioned protein kinases (1:1000, Cell Signaling Technology, USA) and alpha-tubulin (1:2000, #T6074, Sigma-Aldrich, USA). The ratios Bax/alpha-tubulin and Bcl-2/alpha-tubulin and cleaved caspase-3 (17–19 kDa fragment)/alpha-tubulin were also determined. These ratios in control cells were taken as 1.00. The ratio Bax/Bcl-2 was also calculated; in control cells, it was taken as 100%. Blots were developed with Enhanced chemiluminescence detection Novex ECL HRP Reagent Kit (Invitrogen, Waltham, Massachusetts, USA). The films were scanned on a scanner Canon (CanoScan 8800F). Bio7 was used to quantify the optical densities of the positive bands. 

### 4.7. Statistical Analysis 

Data are given as the means ± SEM. The statistical significance of the differences between the three or more groups of data was assessed by one-way analysis of variance (ANOVA) followed by Tukey’s test for multiple comparisons. The significance of differences between the two groups of data was estimated by Student’s *t* test or paired Student’s *t* test. The differences were considered significant at *p* < 0.05.

## Figures and Tables

**Table 1:** The diminution of rescue rates of insulin in the presence of the inhibitors of the PI3K/Akt and MEK1/2/ERK1/2 signaling pathways and inhibitor of insulin receptor tyrosine kinase.

Sample	Rescue Rates of Insulin	*n*
Without inhibitors	41.65 ± 2.4% **	8
In the presence of LY294002	20.9 ± 6.3% *^,#^	6
In the presence of SL327	19.9 ± 5.6% *^,#^	6
In the presence of BMS-754807	12.1 ± 5.7% ^##^	5

Footnote. Cortical neurons were exposed to inhibitors (or incubated without them) for 30 min, then to 1 µM insulin for 1 h and afterwards to hydrogen peroxide for 6 h. The data are means ± SEM of 5–6 experiments. **^#^** and **^##^**—the differences are significant according to Student’s *t* test as compared to the effect of 1 µM insulin in the absence of the inhibitors, **^#^** *p* < 0.05, ^##^ *p* < 0.01. * and **—the protective effect of insulin is significant, * *p* < 0.05, ** *p* < 0.01.

## Supplementary Materials

Supplementary materials can be found at https://www.mdpi.com/1422-0067/20/15/3702/s1.

## Abbreviations

Akt	protein kinase B
AMPK	AMP-activated protein kinase
CREB	cAMP response element binding protein
ERK1/2	extracellular signal-regulated protein kinase
GSK-3beta	glycogen synthase kinase-3beta
IGF-1	insulin growth factor-1
PI3K	phosphoinositide 3-kinase
ROS	reactive oxygen species
TMRM	tetramethylrhodamine
