# Supplementary figures and images for: The Protective Effect of Insulin on Rat Cortical Neurons in Oxidative Stress and Its Dependence on the Modulation of Akt, GSK-3beta, ERK1/2, and AMPK Activities
Source: Int J Mol Sci. 2019 Jul 29;20(15):3702. doi: 10.3390/ijms20153702 (PMC6696072; doi:10.3390/ijms20153702)

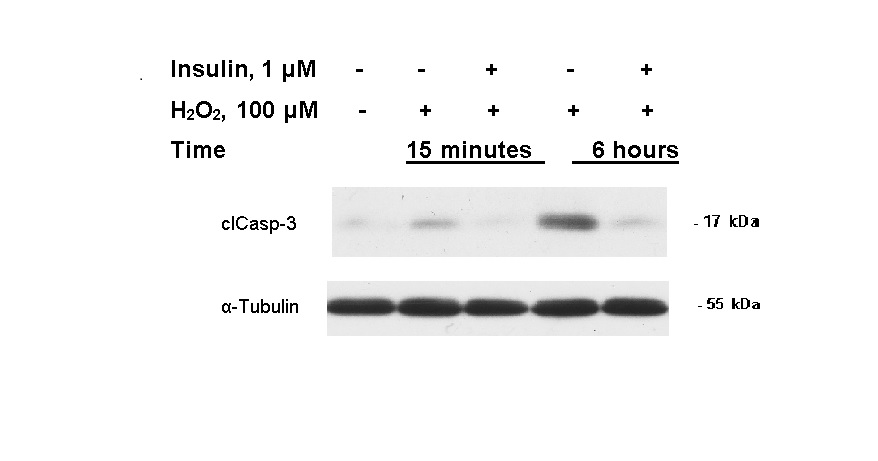

Supplement: Supplementary file 1 [file ijms-20-03702-s001.zip › Supplemental/Figure S1.jpg]

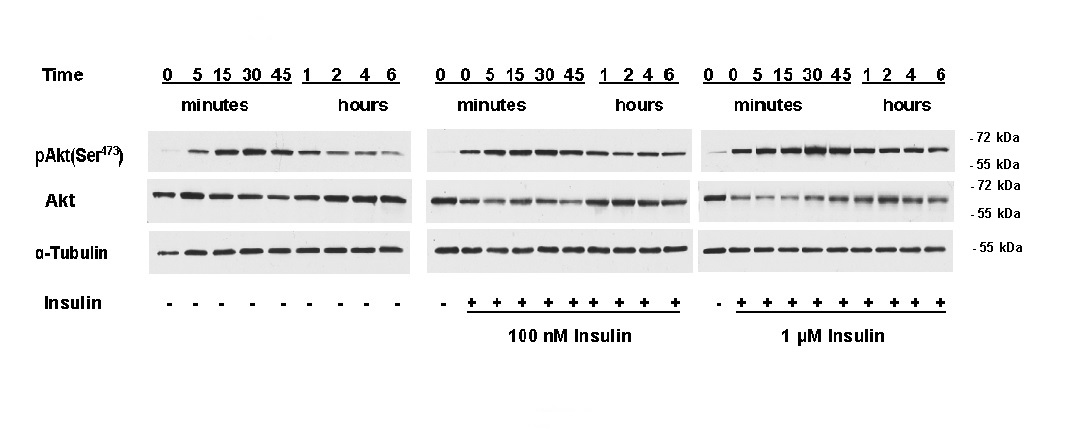

Supplement: Supplementary file 1 [file ijms-20-03702-s001.zip › Supplemental/Figure S2.jpg]

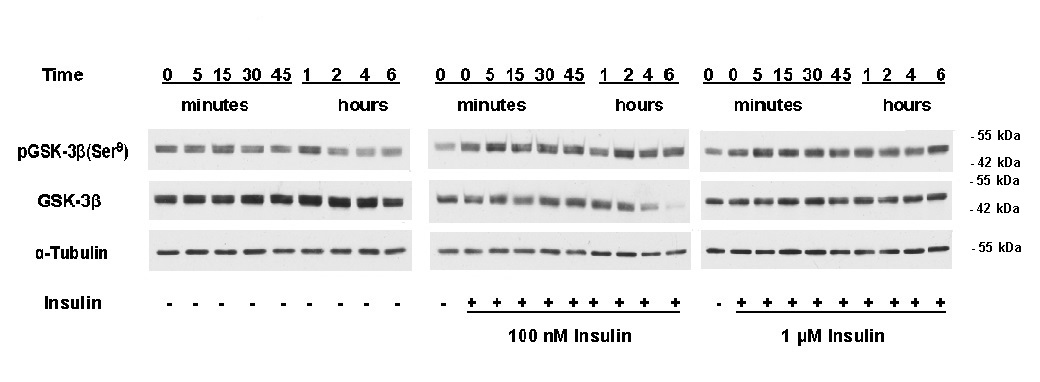

Supplement: Supplementary file 1 [file ijms-20-03702-s001.zip › Supplemental/Figure S3.jpg]

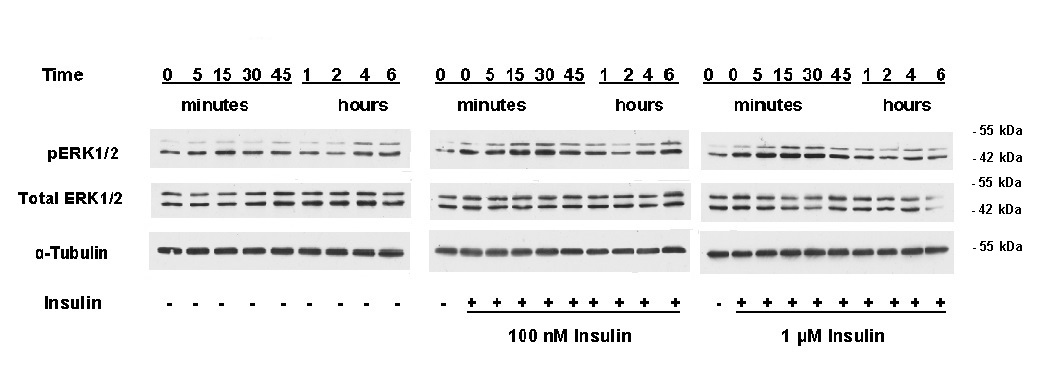

Supplement: Supplementary file 1 [file ijms-20-03702-s001.zip › Supplemental/Figure S4.jpg]

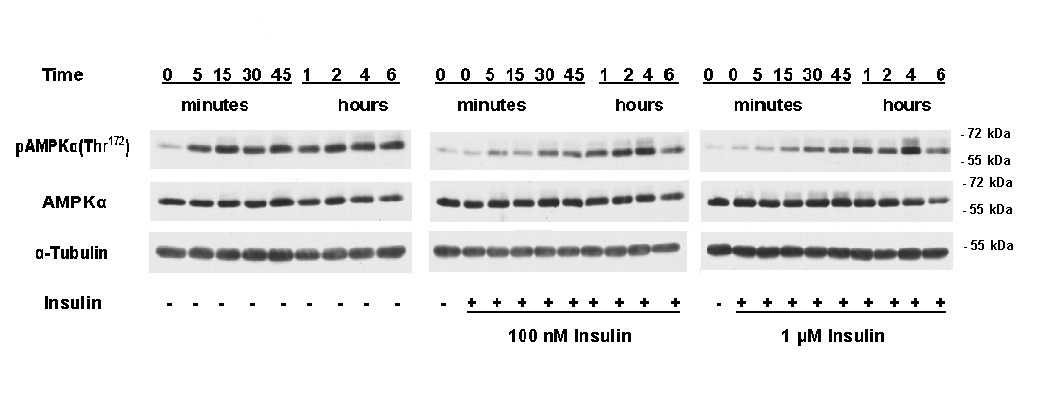

Supplement: Supplementary file 1 [file ijms-20-03702-s001.zip › Supplemental/Figure S5.jpg]

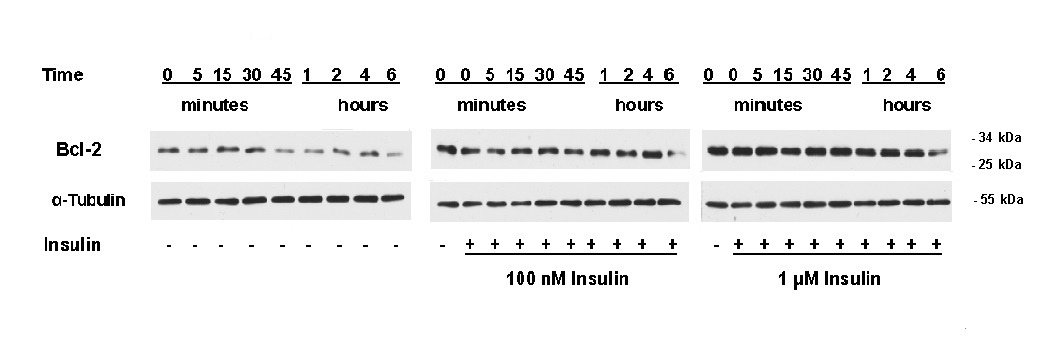

Supplement: Supplementary file 1 [file ijms-20-03702-s001.zip › Supplemental/Figure S6.jpg]
